# Redox-sensitive probes for the measurement of redox chemistries within phagosomes of macrophages and dendritic cells^☆^

**DOI:** 10.1016/j.redox.2013.09.002

**Published:** 2013-09-27

**Authors:** Dale R. Balce, Robin M. Yates

**Affiliations:** a Department of Comparative Biology and Experimental Medicine, Faculty of Veterinary Medicine, Calgary, AB, Canada T2N 4N1; b Department of Biochemistry and Molecular Biology, Faculty of Medicine, University of Calgary, Calgary, AB, Canada T2N 4N1

**Keywords:** Redox, Phagosome, Probes, Macrophage, Dendritic cell

## Abstract

There is currently much interest in factors that affect redox chemistries within phagosomes of macrophages and dendritic cells. In addition to the antimicrobial role of reactive oxygen species generation within phagosomes, accumulating evidence suggests that phagosomal redox chemistries influence other phagosomal functions such as macromolecular degradation and antigen processing. Whilst the redox chemistries within many sub-cellular compartments are being heavily scrutinized with the increasing use of fluorescent probe technologies, there is a paucity of tools to assess redox conditions within phagosomes. Hence the systems that control redox homeostasis in these unique environments remain poorly defined. This review highlights current redox-sensitive probes that can measure oxidative or reductive activity in phagosomes and discusses their suitability and limitations of use. Probes that are easily targeted to the phagosome by using established approaches are emphasized.

## Highlights

• A review of redox probes and their use in macrophage and dendritic cell phagosomes.
• Techniques that allow for phagosomal-specific redox measurements are highlighted.
• Advantages and caveats of the most commonly used redox probes are included.

## Graphical abstract

**Figure:**
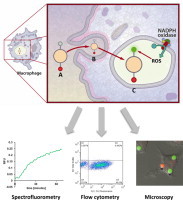

## Introduction: redox systems in phagosomes

Phagosomes are vacuolar organelles resulting from the engulfment of extracellular material by specialized immune cells called phagocytes, such as macrophages and dendritic cells. The resultant organelle goes through a series of fusion and fission events with endosomes and subsequently lysosomes to produce a hybrid organelle – the phagolysosome. Throughout the phagosome's maturation, it accumulates antimicrobial and degradative components that serve to kill and digest phagocytosed material such as microbes [1]. The production of reactive oxygen species (ROS) within phagosomes by NADPH oxidase (NOX2) has been shown to be a major contributor to the antimicrobial function of this organelle, and has been studied extensively [2]. This contribution is highlighted in patients with chronic granulomatous disease in which the absence of functional NOX2 leads to recurrent infections [3]. Whilst much of the field has focused on the oxidative characteristics of the phagosome, the reductive capacity of the phagosome has received less attention. Nonetheless, increasing evidence indicates that reductive chemistries within the phagolysosome play critical roles in phagosomal function and homeostasis [4,5]. For instance, enzymes capable of reducing disulfide bonds are required for the efficient processing of disulfide-bond-containing protein antigens within the acidic phagosomal lumen [6]. Furthermore, oxidation-sensitive proteases, such as the lysosomal cysteine cathepsins, require a reducing environment for activity [7,8]. Rybicka et al. have recently shown that ROS production by NOX2 negatively regulates both disulfide reducing mechanisms and thiol-dependent proteases in the phagosome [4,9]. As factors that affect antigen processing and subsequent presentation to the adaptive immune system have major implications in a variety of pathological conditions, phagosomal redox chemistries may be of great significance to health and disease. Surprisingly, very little is known about the mechanisms that control and maintain the redox microenvironment within these compartments. A major impediment to this line of research has been the lack of convenient technologies that can accurately measure redox chemistries within the phagosome. In this review current techniques and probes that are used to measure oxidative and reductive events in the phagosomes of macrophages and dendritic cells are highlighted.

## Approaches: measurement of phagosome-specific redox chemistries

In order to accurately assess redox events in the phagosome, ideal probes must be highly sensitive and compartment-specific, and should allow for both temporal and spatial resolution. In addition they must be specific to the type of redox event being measured, and resistant to interference by other nearby chemistries which are present in the phagosome (such as pH and proteolysis). In the case of oxidative processes, the probes used should ideally be specific for particular ROS. The detection of reductive processes should be similarly specific, as it must be able to distinguish between small molecule activity (*e.g.* cysteine, glutathione) and enzymatic processes. This review focuses on fluorescence-based probes due to their sensitivity and relative ease of detection with conventional laboratory instrumentation. As will be discussed in this review, many reagents can measure redox changes with high sensitivity, however not all are suitable for use within the phagosomal lumen.

**Fig. 1:**
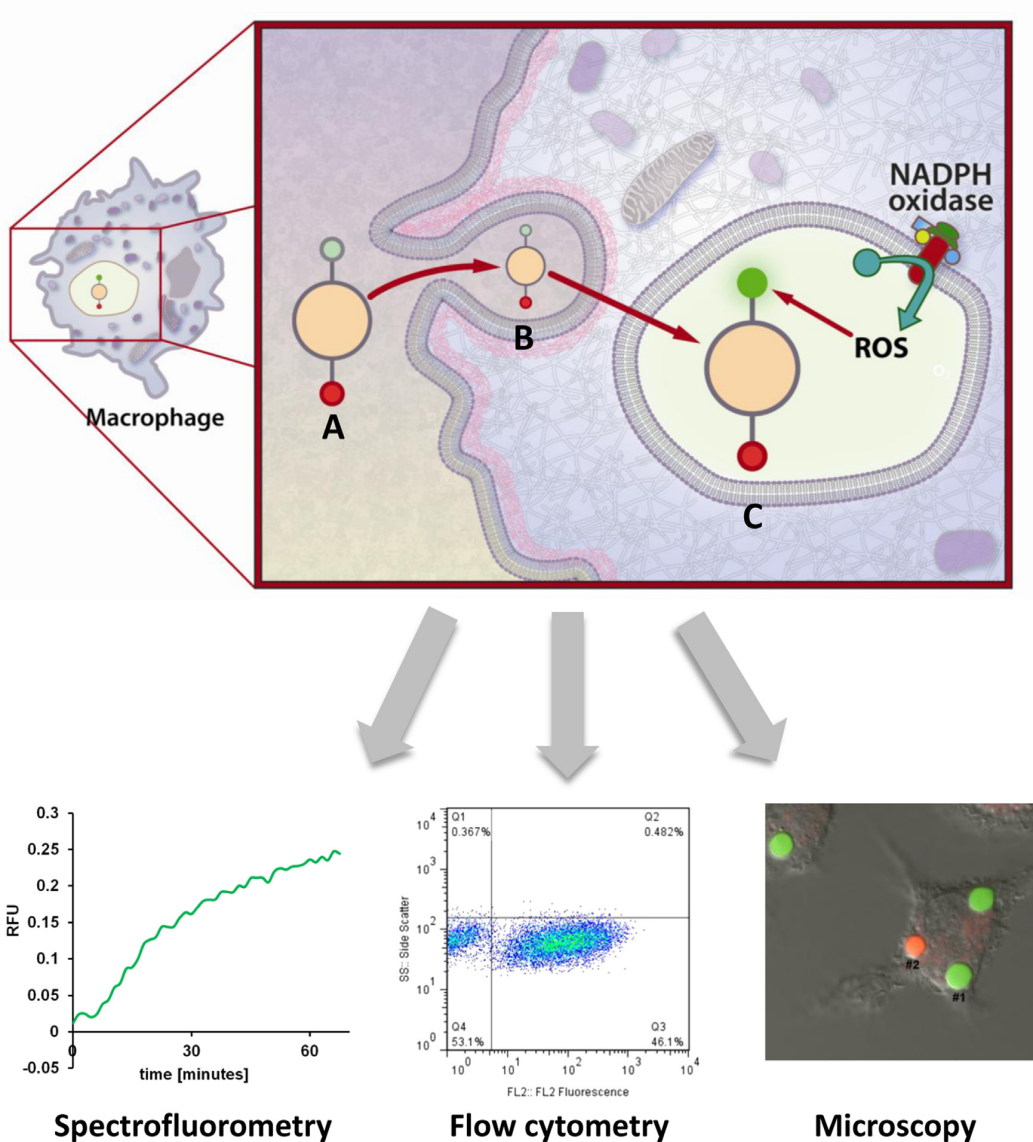
(A) Experimental particles bearing the redox-sensitive probe (green) and the calibration fluorophore (red) are targeted to phagocytes. (B) The experimental particle is internalized via receptor-mediated phagocytosis through conjugation of receptor ligands to the particle surface. (C) After internalization, the resulting phagosome acquires oxidative/reductive capacity (in this figure the generation of ROS is used as an example). Fluorescence of the probe increases while fluorescence of the calibration fluorophore remains the same. Ratiometric measurements can be acquired by fluorometry, fluorescence microscopy or flow cytometry. (For interpretation of the references to color in this figure legend, the reader is referred to the web version of this article.)

A common approach utilizes redox-sensitive fluorescent probes covalently- or non-covalently coupled to experimental particles, which are subsequently given to phagocytes [10–12]. Since each probe is directly conjugated to an engulfed particle, this approach allows for the reliable measurement of phagosome-specific activity. By synchronizing the phagocytosis of the particles (*e.g.* by a short pulse followed by removal of extracellular particles), phagosomal parameters can be measured throughout their maturation in a time-resolved manner. Typically, the experimental particles used are 1–3 µm in diameter and are composed of polystyrene latex or silica, to which probes are conjugated through functionalized surface chemistries (typically –NH_2_ or –COOH). 3 µm silica particles are preferred in the Yates lab as they are dense (easy to synchronize as they quickly fall onto cellular monolayers) and generally have a higher availability of surface chemistries that can be used for conjugation. In addition to the redox-sensitive fluorophore or substrate, a second fluorophore which is insensitive to the chemistries within the phagosome is usually conjugated to the particles for calibration purposes. It is also possible to manipulate the mode of uptake by conjugating various phagocytic receptor ligands to the experimental particles [9]. Following phagocytosis, fluorescence can be monitored in a population-based format using a spectrofluorometer or fluorometric plate reader, or in a single cell-based format using confocal microscopy or flow cytometry (Fig. 1) [11].

Fluorometers are well suited for determining population-based differences in phagosomal redox activity [11]. The latest generation of fluorescence plate readers allows rapid read times for high temporal resolution, and can be equipped with a plethora of optical options/combinations to allow measurement of fluorescence lifetime and fluorescence polarization in addition to standard fluorescence intensity-based analysis. Multi-well plate readers are also easily adaptable for high-throughput analysis [4]. Whilst plate and cuvette-based fluorometers offer highly sensitive and robust readouts from populations of cells, phagosomal or cell heterogeneity is not able to be assessed. To assess these attributes, quantitative fluorescence microscopy can be employed [9,11,13]. The ability to visualize samples also reduces background signal, as well as potential artifacts such as extracellular experimental particles that can occur in fluorometry- and cytometry-based assays. Although the sample throughput, dynamic range and temporal resolution of this modality are typically modest, the ability to determine differences between phagosomes within single cells or those of different cells makes this approach popular in many studies [14]. Another approach that allows phagosomal heterogeneity between cells (but not within cells) is flow cytometry. Cytometry-based analyses can also quickly determine population-based differences in phagosomal redox activity [15,16]. However since sample preparation for these assays are laborious (often require fixing, counter-staining and numerous wash steps), temporal resolution is low and there is a potential for artifacts being introduced between the biological redox event and the time of measurement. Another major drawback of cytometry-based approaches is the low sensitivity of fluorescent detection, which makes the resolution of subtle differences between samples difficult.

A drawback to the approaches outlined above, is that since the majority of commonly available redox-sensitive probes are not specific, they cannot precisely identify the particular redox products being produced (as discussed in more detail elsewhere [17,18]). A complementary approach would be to utilize HPLC coupled with mass spectral analysis of the probes, which has been shown to identify specific redox modifications in combination with the real time assays outlined here [19,20].

## Measuring phagosomal ROS production

NOX2 is the prototypical phagocyte oxidase and the first identified enzyme that creates ROS as an intentional product [21]. NOX2 consists of the cytosolic components, p67, p47, p40 and the small GTPase RAC1/2, which upon activation form a complex with the membrane bound proteins, p22 and gp91 [22]. The assembly of NOX2 on the phagosome can be induced by a variety of stimuli such as Fc receptor activation during the phagocytosis of IgG-opsonized particles [23,24]. Expression of NOX2 in professional phagocytes is induced during differentiation and can also be increased following exposure to activating stimuli such as interferon gamma [25]. The production of ROS in phagosomes begins with the generation of superoxide. Superoxide can dismutate spontaneously or through the enzyme superoxide dismutase, resulting in hydrogen peroxide. Further transformation of these oxidants either enzymatically or through the interaction with metals or reactive nitrogen species lead to downstream reactive species including hydroxyl radicals, hypochlorous acid (in neutrophils) and peroxynitrite [21,26].

**Fig. 2:**
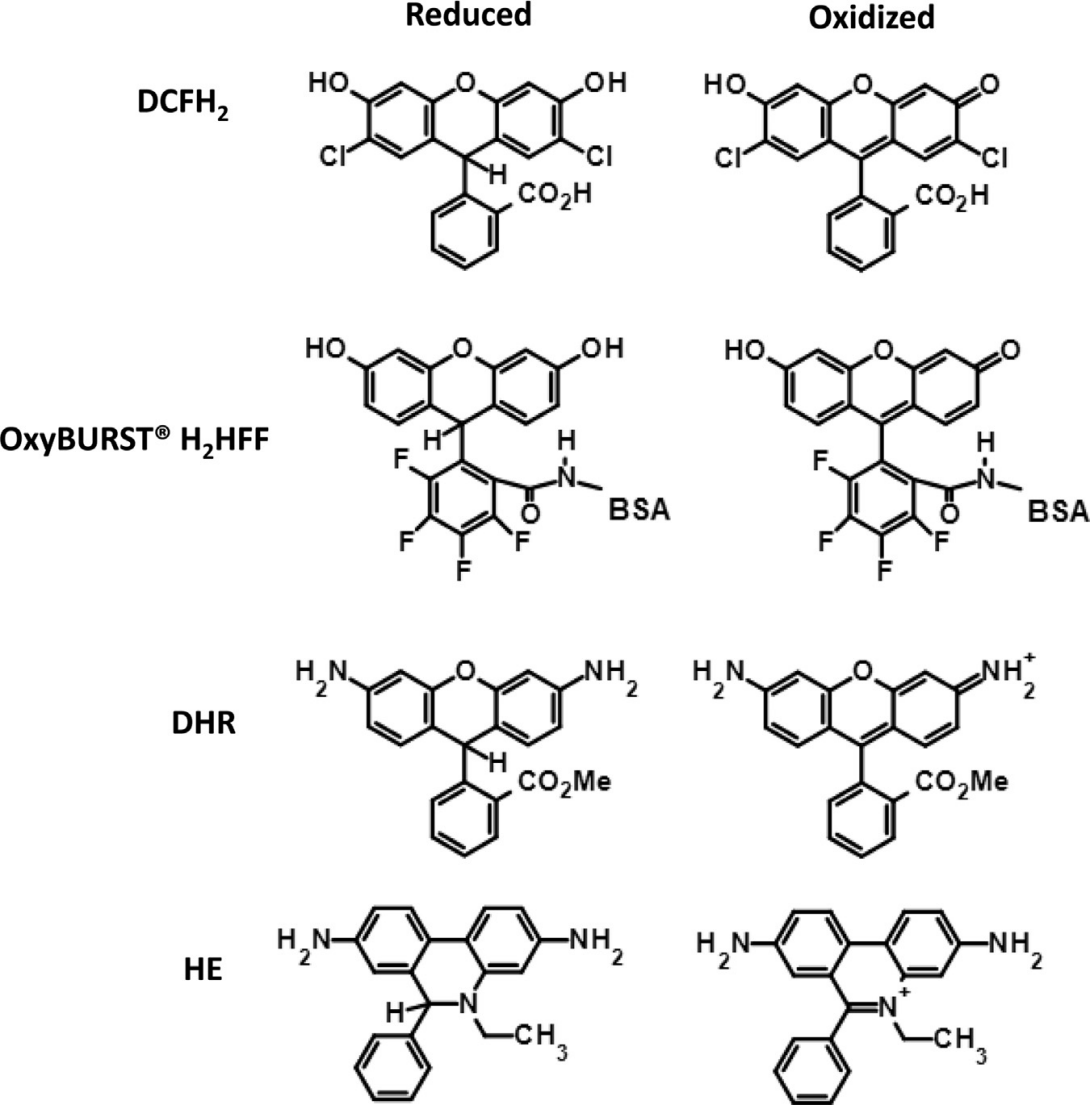
Structures of the dihydrofluorescein derivatives Dichlorodihydrofluorscein (DCFH_2_) and OxyBURST H_2_HFF, Dihydrorhodamine (DHR) and Hydroethidine (HE).

Chemiluminescence-based techniques were some of the first assays used to study ROS production in cells [27]. Lucigenin and luminol are commonly used reagents for ROS detection; however the lack of specificity to individual oxidants and susceptibility to interfering chemistries, as well as difficulties in determining spatial resolution, limit their use in studying phagosomal ROS production [28]. More recently fluorescence-based techniques have become more popular due to their sensitivity and relative ease of detection. Dichlorodihydrofluorescein (DCFH_2_), dihydrorhodamine (DHR) and hydroethidine (HE), which are reduced derivatives of commonly used fluorophores, have been the most popular fluorescent probes used to measure ROS [29]. Oxidation of the reduced fluorophore results in structural changes, leading to increased fluorescence (Fig. 2). Although these reagents can detect various oxidants, the ability to target these reagents to specific cellular compartments often limits their usefulness [13]. For example, HE is highly specific for superoxide, however the oxidized product has a high affinity for DNA and thus accumulates in the nucleus [30]. DHR is membrane permeable both in its oxidized and reduced states and thus it gives no information on the specific location of ROS generation [31], unless it is combined with microscopy [32]. DCFH_2_ is membrane impermeable, thus making it difficult to study intracellular ROS production. The diacetate derivative of DCFH_2_ is membrane permeable, localizes to the cytoplasm and is then cleaved by cellular esterases and retained in the cell. The oxidants detected by this fluorophore would thus be limited to those which can freely diffuse through the phagosomal membrane to the cytosol.

As discussed above, in order to study phagosomal specific oxidative events these probes must somehow be directly bound to phagocytic cargo. Phagosomes can be specifically targeted by latex particles coated with DHR, which can subsequently be measured by flow cytometry and other methods [33,15]. Similarly the succinimidyl ester precursors of DCFH_2_ allow conjugation to amines present on phagocytic cargo for phagosomal specific ROS detection [34]. The Yates lab prefers the use of a modified reduced fluorescein probe conjugated directly onto experimental particles as described by VanderVen et al. [11]. OxyBURST Green H_2_HFF BSA (Invitrogen), which consists of bovine serum albumin coupled to a reduced fluorescein derivative (dihydro-2′,4,5,6,7,7′-hexafluorofluorescein, H_2_HFF), is crosslinked to 3 µm carboxylated silica particles using the heterobifunctional crosslinker cyanamide. Upon oxidation the H_2_HFF compound emits fluorescence at 488 nm excitation/520 nm emission. This compound allows sensitive and accurate measurements of phagosome-specific ROS production in live phagocytes [4,9,11,35]. The OxyBURST Green H_2_DCFDA succinimidyl ester can be used in a similar fashion by direct conjugation to protein coated particles, although it has limited use as the fluorescence of the oxidized product is diminished at low pH. VanderVen et al. also designed another phagosome-specific assay using the lipid peroxidation sensor BODIPY 581/591C11 (Invitrogen). The BODIPY core in the derivatized 11 carbon fatty acid is substituted with a phenyl group through a conjugated diene which is oxidation sensitive. Once oxidized, the phenyl group is freed and the emission spectra maximum of the BODIPY core is shifted from 595 nm to 520 nm. When incorporated into a mixed lipid monolayer of IgG opsonized particles, this probe also allows phagosomal-specific measurement of the oxidative burst through ratiometric imaging in live cells using confocal microscopy.

One major limitation of all the probes described above is that they do not distinguish between different types of oxidants. For example, DCFH_2_ is mainly sensitive to H_2_O_2_ in the presence of a catalyst such as a peroxidase, but it is also oxidized, albeit to a lower extent, by hydroxyl radicals, HOCl and indirectly by peroxynitrite [31,36,37]. DHR has very similar characteristics to DCFH_2_ but differs in that it is more sensitive to oxidation by HOCl. Similarly the OxyBURST Green reagents described above are likely primarily sensitive to H_2_O_2_ in the presence of a catalyst, but the specific oxidant(s) detected by these reagents are currently undefined. Surprisingly DCFH_2_ and DHR derivatives are relatively insensitive to superoxide. HE is highly sensitive to superoxide but can also be oxidized by H_2_O_2_ in the presence of a catalyst [38,39]. Furthermore, HE can be oxidized to different products depending on whether it is oxidized by superoxide or by other oxidants. HPLC coupled with mass spectral analysis can determine which oxidized form of HE is being produced and thus the specific oxidant being measured [19].

To deal with the issue of non-specificity to ROS species, newer probes that measure the nucleophillic nature of the superoxide anion rather than its oxidative properties have been developed. These probes incorporate leaving groups that when cleaved yield the free fluorescent molecule [40,41]. In addition fluorophores such as aminophenyl fluorescein and hydroxyphenyl fluorescein are more specific to downstream oxidants such as HOCl and the hydroxyl radical. Amplex Red is a highly sensitive fluorophore that specifically measures H_2_O_2_ in the presence of a catalyst [42]. Amplex Red, which is a reduced derivative of the highly fluorescent fluorophore resorufin, reacts specifically with H_2_O_2_ in a 1:1 stoichiometry. Quantitative measurements can be made in the picomolar range by regression to a standard curve. Further characterization and incorporation of these probes into phagosome specific assays will provide novel and more specific tools to study the phagosomal production of specific types of oxidants.

## Measuring phagosomal reductive capacity

In comparison to other organelles, mechanisms that control reductive events in the phagosome are less characterized. Disulfide reduction within lysosomes is unlike that in other cellular compartments, as enzymatic catalysis seems to be necessary due to the acidic environment [43]. Hence, whilst low molecular weight monothiol reductants such as glutathione and cysteine can readily reduce substrates at neutral pH, this mechanism is not favored at lower pH [44]. This would suggest the requirement of catalytic enzymes for efficient reductive activity in lysosomes and matured phagosomes. So far, the only lysosomal reductase characterized to date is gamma-interferon-inducible lysosomal thiol reductase (GILT, also known as IP-30). GILT was originally discovered as an interferon gamma inducible gene in the monocytic cell line U937 [45]. Arunachalam et al. further characterized GILT, and identified the presence of a typical redox active Cys–X–X–Cys motif that is present in thioredoxin and other cellular reductases [6,46]. GILT is optimally active at low pH and is targeted to the lysosomal system through the mannose-6-phosphate receptor pathway. Antigen processing and presentation models have been used to characterize the activity of GILT [47,48]. These experiments have generated much information on the ability of GILT to reduce certain model antigens that contain disulfides, such as hen egg lysozyme. Disulfide reduction has been shown to occur in the absence of GILT, suggesting the presence of other lysosomal/phagosomal reductive mechanisms. (For an in depth review on GILT and its role in antigen presentation see Hastings et al. [49].)

As reductive systems such as the thioredoxin system in non-phagosomal compartments have been under study for decades, many tools exist to study disulfide reductase activity (*e.g.* Ellman's reagent). Standard thioredoxin activity kits are also available commercially. Unfortunately these standard assays are not compatible with the acidic, proteolytic microenvironment within the phagosome and thus cannot be easily adapted to study phagosomal disulfide reduction. Antigen presentation assays, such as those used in characterizing GILT, have been successfully used to indirectly evaluate the ability of phagosomes to reduce disulfides within model antigens [5,47,50]. However, these immunological assays do not provide a clear picture of the time, rate and location of the reduction events, and thus are limited in their ability to more precisely investigate mechanistic detail.

**Fig. 3:**
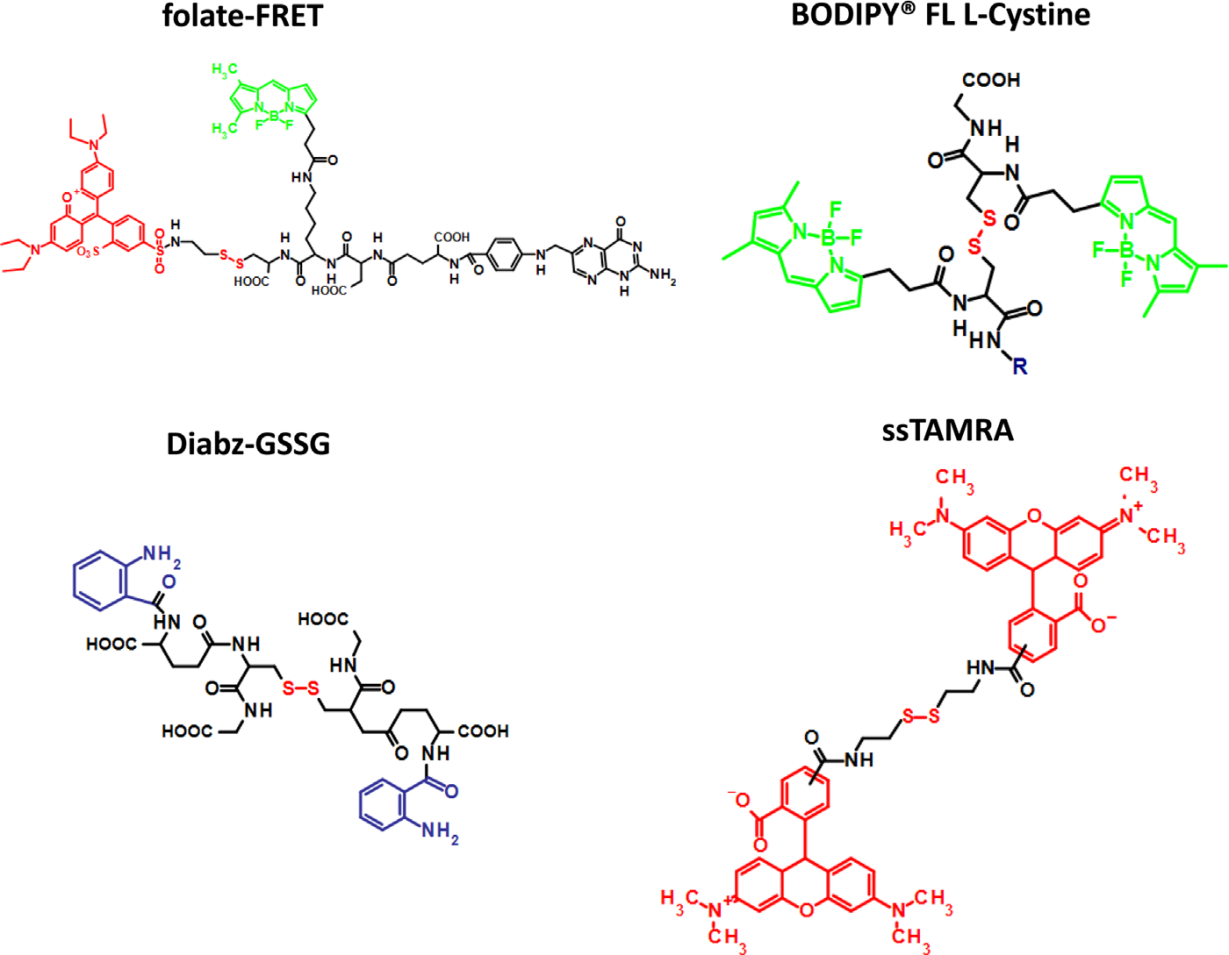
Structures of the folate-(BODIPY FL)-SS-rhodamine reporter (folate-FRET), BODIPY FL l-Cystine, di-(o-aminobenzoyl) glutathione disulfide (diabz-GSSG) and the TAMRA disulfide dimer (ssTAMRA).

To address this need, several fluorescence-based techniques have been developed to study disulfide reduction in endosomes and lysosomes (ELs) (phagosome-associated vacuolar organelles). In order to measure EL disulfide reduction, Austin et al. conjugated rhodamine red to their disulfide linked antibody-drug compound at a labeling ratio capable of causing the rhodamine red to self-quench [51]. Although they showed efficient dequenching of the conjugate with artificial reductants *in vitro*, they showed that ELs were not capable of reducing the disulfide linked antibody drug conjugate. This observation was in agreement with others who had previously reported that endosomes were more oxidizing than reducing [44]. In contrast, Yang et al. developed a Förster/Fluorescence Resonance Energy Transfer (FRET) based probe to show that ELs were indeed competent in disulfide reduction [52]. The folate-(BODIPY FL)-SS-rhodamine reporter consisted of folic acid to target folate-receptor-mediated endocytosis and a peptide spacer to which the flurophores were conjugated. The donor fluorophore in the FRET pair was BODIPY FL, and the acceptor fluorophore was tetraethyl rhodamine which was linked to the peptide spacer via a reducible disulfide linker (Fig. 3); thus a loss of FRET would be observed upon reduction. The FRET signal was measured by both flow cytometry and fluorescence confocal microscopy. Although this group specifically measured endosomal reductive events, this probe could potentially be modified for phagosome-specific measurements by changing the receptor ligand or through conjugation to larger particles. The discrepancies between the two studies described above could be due to a number of factors. One possibility is that the redox environment within ELs can vary depending on the route of cargo intake (*i.e.* IgG vs. folate receptor). Another possibility is that certain probes, although designed around simple disulfide linkers and cleavable by artificial reductants *in vitro*, may not be recognized by the enzymatic systems in ELs such as GILT due to conformational or steric restraints.

Probes for disulfide reduction based on self-quenching fluorophores linked by reducible disulfides are gaining popularity. Since they rely on self-quenching properties, their design and synthesis is simpler than their FRET-based counterparts. The Yates group recently developed an assay using BODIPY FL l-cystine (Invitrogen), which was the first of its kind to specifically and accurately measure phagosomal disulfide reduction in real-time in live cells [4,9,35]. The reporter in this assay is a substrate that consists of two BODIPY FL fluorophores which are self-quenched when linked by the disulfide-containing cystine molecule. Upon reduction, BODIPY FL emission can then be measured as the fluorophores dequench. This substrate is efficiently reduced by lysosomal lysates [9] as well as recombinant thioredoxin and GILT in reconstituted systems (unpublished data). To measure rates of disulfide reduction within phagosomes of live cells, this substrate can be covalently linked to experimental particles and fed to phagocytes. This can be achieved through coupling the free carboxyl group present within cystine to either protein or amine modified dextran-coated experimental particles using carbodiimide-mediated coupling in the presence of N-hydroxysuccinimide [4]. The use of this reporter revealed that phagosome-specific reductive capacity is highly dependent on the extent of NOX2 activation immediately following internalization of phagocytic cargo [4,9,35]. Since NOX2 assembly is highly influenced by ligands on phagocytic (or endocytic) cargo, this might provide insight into the discrepancies between earlier studies in ELs [24,53,54].

Other promising self-quenching probes which could potentially be used to study phagosomal reductive events include Diabz-GSSG (di-(o-aminobenzoyl) glutathione disulfide) and ssTAMRA (a disulfide containing tetramethylrhodamine (TAMRA) dimer) (Fig. 3). Diabz-GSSG, developed by Raturi et al., consists of isatoic anhydride conjugated to oxidized glutathione [55]. A similar oxidized glutathione-based probe was also developed by the same group using eosin as the self-quenching fluorophore [56]. These probes were shown to be reduced by protein disulfide isomerase *in vitro*, resulting in measurable dequenching of the fluorophores [55]. Using a similar strategy, Christie et al. utilized an oxidized dimer of the fluorophore TAMRA as a probe for disulfide reduction. Since the spectra of the reduced monomeric TAMRA and ssTAMRA are distinct, reduction of the dimer can be monitored by calculating absorbance ratios of 554 nm (reduced) over 520 nm (oxidized). In addition, relative fluorescence is nearly extinguished in the oxidized dimer due to self-quenching. The authors demonstrated that ssTAMRA is highly sensitive to dithiothreitol as well as physiological reductants present in cell lysates from RAW macrophages [57]. Further characterization of these probes and development of derivatives that allow conjugation to experimental particles could lead to novel tools to study phagosomal reductive mechanisms.

## Future directions of phagosomal redox-sensitive probes

The reduction potentials (in millivolt values) of endosomes, lysosomes and phagosomes are still unknown. One major drawback is that the redox buffer system within the phagosome is currently unclear. Cysteine/cystine and/or reduced glutathione/oxidized glutathione may play this role, but how these systems are established and maintained (for example the identification of phagosomal specific membrane transporters) remains controversial [8,43]. With the development of more sensitive and reversible redox probes, the dynamics of phagosome reduction potential may be elucidated in the near future. The redox probes discussed above are limited to one oxidation/reduction event, thus suffer from substrate limitation. Reversible redox probes would allow dynamic changes in reduction potential to be studied throughout phagosomal maturation. Miller et al. have recently incorporated a disulfide onto a fluorescein scaffold resulting in a probe sensitive to continuous oxidation and reduction events [58]. Similar reversible probes have also been recently developed [59,60]. There has also been a surge in the development of redox-sensitive fluorescent protein (roGFPs) in recent years, which may help elucidate the reduction potentials of endo/lyso/phagosomes. Due to their susceptibility to proteolysis and changes in pH, the use of roGFPs in phagosomes may be limited. Nonetheless newer variants of roGFPs have been very promising due to their sensitivity to both oxidative and reductive events, and their ability to be targeted to specific cellular compartments [61–63].

## Redox considerations for other phagosomal probes

The use of fluorescent probes to measure phagosomal parameters such as pH and enzymatic activities in live cells is gaining in popularity [10,15,16]. Coupled with the ever-increasing number of commercially available fluorescent probes, numerous parameters of the phagosomal microenvironment can be studied in live cells. However, caution must be used when determining the suitability of probes for use within the phagosome where several potentially interfering chemistries occur in a temporally- and spatially-intimate fashion. In addition to fluorophore function at low pH and susceptibility to hydrolysis, the potential for probe oxidation should also be considered. An example of this is the use of the newer generation pH sensors pHrodo (Invitrogen) and cypHer (GE) for the measurement of phagosomal pH. Unlike more traditional fluorescent sensors, such as carboxyfluorescein and Oregon green, these fluorophores have red to far-red emission and gain-in-fluorescent properties with lowering pH. Moreover these fluorophores have been marketed as indicators of phagosomal acidification. Unfortunately, both of these fluorophores are sensitive to oxidation by ROS. Specifically, Rybicka et al. have shown that NOX2 activity markedly and irreversibly alters the relationship between probe fluorescence and pH within the macrophage's phagosome [9]. This could potentially lead to grossly disparate measurement of pH between samples which differ only in NOX2 function, making these probes unsuitable for pH determination within this compartment. This highlights the need for careful characterization of each probe to identify and limit potential artifacts caused by interfering chemistries present in the phagosome. In addition to pH and hydrolysis, redox-modification to probes should be considered in determining probe suitability for phagosomal measurement.

## Concluding remarks

Although redox-sensitive probes are widespread, specific limitations preclude many of them to the study of phagosomes. Nonetheless, recent advent of phagosomal specific probes and assays have enabled the investigation of basic redox chemistries within the phagosome and their role in phagosomal function.
